# A Mixed-Methods Study on Use of Different Tobacco Products among Younger and Older Adults with Lower and Higher Levels of Nicotine Exposure in California in 2019–2020

**DOI:** 10.3390/ijerph19095563

**Published:** 2022-05-03

**Authors:** Arit Harvanko, Kimberly A. Koester, Gideon St. Helen, Sarah Olson, Hyunjin Cindy Kim, Pamela M. Ling

**Affiliations:** 1 Center for Tobacco Control Research and Education, University of California, San Francisco, CA 94143-1390, USA; aritharvanko@hotmail.com (A.H.); kimberly.koester@ucsf.edu (K.A.K.); gideon.sthelen@ucsf.edu (G.S.H.); hyunjin.kim2@ucsf.edu (H.C.K.); 2 Divison of General Internal Medicine, Department of Medicine, University of California, San Francisco, CA 94143-1390, USA; sarah.rosen@ucsf.edu

**Keywords:** ENDS, e-cigarettes, cigarettes, nicotine gum, TNE, mixed methods, pod

## Abstract

An increasing number of tobacco products are entering the market, offering a variety of options to attain desired nicotine intake. However, little is known about the effect of this diverse marketplace on the experiences and nicotine exposure among tobacco users. A mixed-methods study examined experiences with tobacco products among individuals with relatively lower or higher levels of biomarkers of nicotine exposure. Semi-structured interviews were conducted with younger and older adults to examine tobacco use behaviors, addiction to tobacco products, and nicotine effects. Younger and older adults provided similar narratives about addiction and nicotine effects, which were similar across age groups, product types (i.e., ENDS, combustible cigarettes, or oral tobacco products), and having lower or higher nicotine exposure. Some individuals with higher nicotine exposure who switched from combustible cigarettes to another product (e.g., ENDS) found similar or greater access and perceived addiction to nicotine. Adults with higher and lower levels of nicotine exposure provided narratives consistent with features of nicotine addiction, regardless of age and products used. Availability of multiple tobacco products may be associated with greater access and exposure to nicotine. Addiction may occur at low levels of use and with non-cigarette products.

## 1. Introduction

In recent years the tobacco product landscape has become more diverse, with new additions to the marketplace such as oral nicotine products (i.e., nicotine-containing pouches [1]), different types of electronic nicotine delivery systems (ENDS) (e.g., refillable or disposable pod-type devices [2]), heated tobacco products (e.g., Phillip Morris’ IQOS [3]), and others. Many tobacco products are marketed as alternatives to combusted cigarettes [4,5], and individuals may incorporate these products into their daily routines in addition, or as an alternative, to combusted cigarettes. In 2019, an estimated 50.6 million U.S. adults (20.8%) reported currently using any tobacco product, including cigarettes (14.0%), ENDS (4.5%), and smokeless tobacco (2.4%), and 18.6% of tobacco users reported using two or more tobacco products [6]. As more nicotine-containing products enter the marketplace, it is possible that a growing number of individuals will switch from combustible cigarettes (either temporarily or long-term) or continue combustible cigarette use in addition to the use of other products. In California, where this study took place, there are strong and longstanding tobacco control policies [7]: a USD 2 increase in tobacco taxes enacted in 2018; over 94% of the population is covered by local smoke-free policies, and 79% of the population is covered by policies regulating retail tobacco sales. Smoking prevalence has declined to 6.9% among adults in 2019 and 1.2% among high school youth in 2020 [8], and multiple tobacco product use has become increasingly relevant: with past-30 day use of ENDS reported by 5.1% of adults in 2019 and 8.2% of youth in 2020 [8].

Tobacco product switching and co-use have been difficult to research due to the rapidly changing tobacco product landscape. It is necessary to assess the impact these products have on users, particularly motivations to use and associated experiences, to better understand the effects of new products on nicotine exposure, dependence, and disease risk. For example, individuals using or switching between multiple different types of products may achieve nicotine intake that is equal to or greater than single product use. Greater nicotine exposure could occur because different products can allow individuals to consume nicotine more discreetly in places where smoking is prohibited [9], or discreet products may be used more easily by individuals under the legal age for tobacco consumption [10]. New tobacco products may have less aversive characteristics (e.g., the presence of ashes or smoke [11]) or deliver comparable amounts of nicotine as other products at a lower price point or with greater convenience [12]. As such, it is necessary to examine how the use of new products affects tobacco use behaviors, nicotine exposure, and symptoms and perceptions of dependence.

As the use of multiple tobacco products increases, it can be difficult to track and quantify nicotine intake among this population using self-report measures. This is because, with regards to nicotine exposure, there is not a standard equivalency for different types of tobacco products (e.g., ENDS, combustible cigarettes, and oral tobacco products). A further difficulty arises with products like ENDS because there are often user-adjustable parameters (e.g., liquid nicotine concentration) that can affect nicotine exposure [13]. Nicotine and its metabolites can serve as objective measures of nicotine exposure across users of different types of tobacco products [14]. Biomarkers of nicotine exposure also make it possible to compare individuals who use different types of tobacco products as a function of nicotine exposure.

The purpose of the current study was to utilize a mixed-methods approach to detail how younger and older adult tobacco users utilize different products in their daily routines. It was hypothesized that addiction narratives, perceptions of products, and motivations for product use would differ based on levels of nicotine exposure. It was also hypothesized that some products would be associated with differences in addiction narratives and that younger and older adults may differ in the types of tobacco products they use and their motivations for using these products.

## 2. Materials and Methods

Semi-structured interviews were conducted on a sample of younger and older adult tobacco users from one wave of an ongoing longitudinal study on poly-tobacco users (current, past 30-day users of at least two tobacco products). Participants were asked about tobacco use behaviors, including product types, patterns of use, and motivations and perceptions of different products. Participants also provided urine samples that were tested for levels of nicotine and several of its metabolites to estimate recent exposure to nicotine.

### 2.1. Participants

Participants in this study represent a sub-sample of two ongoing longitudinal mixed-method cohorts of poly-tobacco users in California. One cohort consisted of younger adults (18–29 years) recruited in 2017, and the other older adults (30 years and older) recruited in 2018. Eligibility criteria at the first visit included past 30-days use of at least two of the following products: combustible cigarettes, ENDS, and smokeless tobacco. Participant data included in this report are from the second (older adults) and third (younger adults) waves of each respective study, collected between 2019 and 2020. Participants completed an online screening questionnaire to confirm eligibility and were selected purposively at study onset to ensure a diversity of product use patterns and demographic characteristics. Specific recruitment details were previously published [15]. Interviews were conducted primarily in the San Francisco Bay Area, the Central Valley, or Los Angeles in person, although participants who moved or were unable to meet in person due to the COVID-19 pandemic were interviewed by phone. This study reports on a subsample of 47 participants (27 younger adults (aged 20–31 at the time of this study) and 20 older adults (aged 32 years or older at the time of this study)) who remained in the study for the second (older adults) and third (younger adults) wave and consented to provide a urine sample at the time of interview. For this analysis, we included only participants who reported using nicotine in the 3 days preceding the interview to ensure quantifiable levels of nicotine and its metabolites for the biomarker analysis.

### 2.2. General Procedures

Participants completed a baseline questionnaire with validated measures of tobacco product use, attitudes, sociodemographic characteristics, and custom measures of use of newer nicotine and cannabis products. Before each follow-up interview, participants also completed a questionnaire reporting their current frequency of tobacco use. Interviews aimed to capture participants’ experiences, perceptions, and routines of poly-tobacco product use. Interviews were conducted by an interdisciplinary and diverse research team in terms of race, age, gender, disciplinary backgrounds, and experience with qualitative research. The following authors were involved in conducting the majority of the interviews: AH (Ph.D., postdoctoral fellow, novice interviewer), KK (Ph.D., anthropologist, expert interviewer), HCK (MPH, project coordinator intermediate-level interviewer) SR (research coordinator, expert interviewer), and PL (MD, PI, expert interviewer). Two additional postdoctoral fellows also participated in data collection, and each was an intermediate-level interviewer. Team members with little to no prior experience interviewing received extensive training in didactic and experiential formats. For example, novice interviewers shadowed expert interviewers until they felt comfortable conducting interviews on their own. Since the younger adults were repeatedly interviewed, when possible, the same individual who had interviewed them during previous waves of data collection conducted the interview. During interviews, participants described, in a storytelling format, their tobacco product use over the course of a typical weekday. Interviewers prompted participants to describe when, where, how, and why they used a particular tobacco product and why they might prefer it to other products at a given moment of the day. Towards the end of the interview, interviewers asked participants to then quantify all tobacco and cannabis product use during the prior seven-day period using a validated timeline follow-back (TLFB) instrument [16]. They were asked to lead the interviewer through a typical weekend day in a similar fashion. Immediately following each interview, the interviewer completed a field note including observed participant characteristics, behavior, reflections on the quality and character of the interaction, content relevant to specific topics of interest, and questions or curiosities for future data collection. For in-person interviews, participants provided a urinary sample at the time of the interview. For interviews conducted by telephone, urine samples were collected and shipped by the participant, using study-provided packaging and instructions, to the study laboratory. Interviews lasted approximately one hour and were conducted primarily in university seminar rooms or by phone.

### 2.3. Assessments 

#### Nicotine and Cotinine Measures 

Urinary nicotine and metabolites were determined by LC–MS/MS modified for tandem mass spectrometry for improved sensitivity [17]. Total nicotine equivalents (TNE) were calculated by converting nicotine and six of its metabolites (i.e., total (free plus conjugated) cotinine, hydroxycotinine, nicotine oxide, cotinine oxide, nornicotine, and norcotinine) to moles, which were then summed. Unlike biomarkers such as cotinine, TNE is not influenced by metabolism, sex, or diet and, when measured at a steady-state, accounts for 80−90% of a daily dose of nicotine [18].

### 2.4. Data Analysis

The respective data sets were analyzed using an explanatory mixed methods design [19], whereby the qualitative analysis was guided by participant TNE levels and the timeline follow-back report of tobacco use. The qualitative data included the TLFB report, fieldnotes, and full interview transcripts. Quantitative data included participant demographics, tobacco use behaviors, and levels of TNEs.

Participant cases were divided into high and low nicotine exposure groups based on rank-ordered TNE levels to ensure substantial differences in nicotine exposure between groups. We selected five cases of the highest and five of the lowest TNE levels from both younger and older cohorts. Participant demographics, tobacco use behaviors, and levels of TNEs were compared between the younger and older adults, and the low and high nicotine exposure groups, using *t*-tests for continuous variables and chi-squared tests for count variables. Due to their logarithmic distribution, TNE values were log-transformed for analysis. Quantitative analyses were performed using RStudio version 1.0.136 (Rstudio Team (2022). Rstudio: Integrated Development for R. Rstudio, PBC, Boston, MA, USA) and α < 0.05.

Salient information from these qualitative data was extracted and summarized in a template according to key topics of interest, including narratives about tobacco product use, nicotine effects, and perceptions of addiction, which were placed alongside the seven-day TLFB data. Cases were templated by four members of the research team (AH, PL, KK, SR), with each analyst reviewing 6–7 cases. During weekly meetings, high and low nicotine exposure cases were compared, and similarities and differences within and across cases were discussed and documented.

Tobacco product use and subjective experiences from a template analysis of the qualitative interviews were compared to TNE levels for consistency. Data among these consistent cases were integrated, and the cases where the biomarker and interview data were discrepant were analyzed to identify possible explanations for the discrepancy. Presented below is an analysis in which equal emphasis was applied to TNE levels and qualitative interview data.

## 3. Results

**Figure 1:**
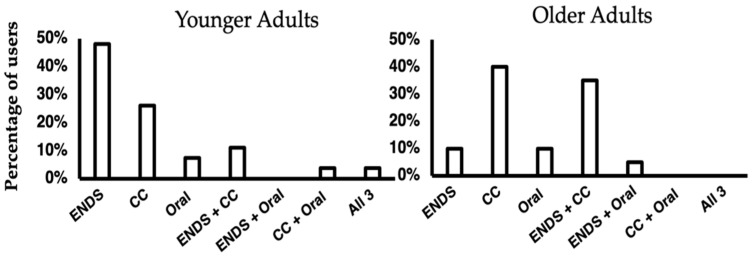
Types of Products Used in the Past 3-days Among the Overall Sample. ENDS = Electronic nicotine delivery system, CC = Combustible cigarette or cigar, Oral = smokeless tobacco or nicotine-containing gum. Significantly more older adults smoked cigarettes or cigars in the past 3-days compared to younger adults (𝜒^2^ = 7.2, *p* = 0.007).

**Figure 2:**
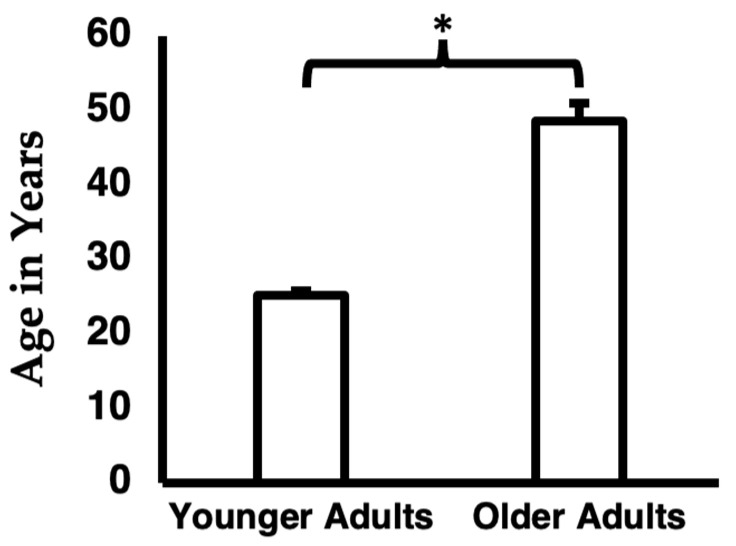
Average Age of the Younger and Older Adult Groups. Error bars represent standard error of the mean. * = *p* < 0.05.

Demographics and tobacco use variables are reported in Table 1 for the overall sample. Expectedly, there was a significant difference in age between the younger and older adult groups (Table 1). Between the younger and older adult groups, there was not a significant difference in the number of participants having used a combusted cigarette or cigar in the past 3-days though, among the younger or older adults that used a combusted cigarette or cigar in the past 3-days, older adults used significantly more combusted cigarettes or cigars in the past 3-days compared to younger adults (Table 1 and evidenced in Figure 1). Demographics and tobacco use variables are reported in Table 2 for the low and high nicotine exposure groups, and there were no significant differences between these groups on demographic or tobacco use variables. TNE levels for the overall sample and low and high nicotine exposure groups are described in Table 3. There was not a significant difference in TNE levels between younger and older adults (*t* = (45)1.6, *p* = 0.114). Expectedly, there was a significant difference in TNE levels between low and high nicotine exposure groups (*t*(18) = 11.4, *p* < 0.001). Categories of products recently used by younger and older adults are shown in Figure 1 and the average age of the younger and older adult groups is shown in Figure 2. 

Initial analysis of the interview data examined younger and older adult groups separately. Results of this analysis indicated the reported nicotine effects and addiction narratives were similar between younger and older adults. Thus, subsequent analysis focused on comparing high and low nicotine exposure groups, combining younger and older adults.

### 3.1. Product Use and Nicotine Effects

Among the 10 participants in the high nicotine exposure group, 4 had used multiple products, 3 used only ENDS, 2 only combustible cigarettes, and 1 participant exclusively used a nicotine-containing gum in the 3 days prior to the urine sample according to the accompanying TLFB. Though nicotine-containing gum is technically not considered a tobacco product, this individual was included in the analysis because the intent of this study was to examine nicotine use from different sources. Although cannabis use was not examined in the biomarker analysis or a systematic focus of qualitative interviews, it is worth noting that five of these cases reported they had smoked or vaped cannabis in the past three days. When the quantity of nicotine used daily was examined, one participant reported smoking a pack of combustible cigarettes a day (this participant also used a box mod to fulfill flavor desire and a JUUL when discretion was needed), two cases used one JUUL pod per day, and all others reported 4–20 cigarettes per day (*m* = 12) and/or 5–60 (*m* = 30) puffs from an ENDS per day. The individual using nicotine-containing gum chewed 6–8 pieces of gum per day. 

Individuals in the high nicotine exposure group described their product use in ways that indicated a strong desire to have continuous access to nicotine (i.e., “I chew constantly” or “I’m trying to get as much nicotine into my body as possible”). However, despite a desire for continuous use, there were reports of relatively minimal nicotine effects. Instead, for many, nicotine effects were associated with the ritual and habit of tobacco use that would lead to relief from a nicotine craving, as exemplified by PT059, who said: “maybe it’s just routine since I’ve been smoking for, for so long”. Others in the high nicotine exposure group described nicotine effects as “satisfying”, as “a reward”, or offering a sense of relaxation or pleasure. For the majority, physical effects such as light-headedness or “head rush” sensations were perceived to have diminished over time. PT059 explains:

*Or someone who has like never smoked before, you smoke a cigarette and you get a little dizzy. Some kind of like little head high or something. I could still do that with the chewing tobacco if I chew enough of it. Um, if I chew enough of it. I, I don’t, but, yeah, you can.*

PT068 echoes this sentiment:

*It used to be was, like, a head rush and sort of, like, the—that woozy feeling. Now for me it’s, um, I’m not sure it’s anything more than just, like, this dopamine trigger… for me it’s like, oh, I’ve accomplished what I want, that goal of wanting to hit the JUUL almost is, like, what it is.*

Relative to each other, participants in the low nicotine exposure group reported similar quantity and frequency of tobacco use on the TLFB, with a few outliers. Two individuals reported using multiple tobacco products in the past three days. Two cases used a tobacco product on an intermittent basis (one ENDS user and another smokeless tobacco user), while all others were daily users of either combustible cigarettes, ENDS, or an oral tobacco product. Note, two of the cases (one exclusive combustible cigarette smoker, one exclusive pod-vape user) in the low nicotine exposure group were identified as “discrepant” in that their biomarker data (e.g., low TNE level) did not align with the subjective reporting of use (e.g., 10 CPD). Because most cases were daily nicotine users, their use patterns could be described as regularly accessing nicotine throughout the day, though less frequently within each day compared to those in the high case category. Nicotine effects among low cases were sometimes described as calming or stress relieving or, similar to those in the high nicotine exposure group, described nicotine as having an energizing effect: “it stimulates my mind a bit” or “it helps me focus a little more, wakes me up a little bit”. Similar to several cases in the high nicotine exposure group, when asked about subjective effects of nicotine, low nicotine exposure group cases also had difficulty conjuring language to depict the physiological sensations and instead explained a lack of effect and more as a routine or habit. This was exemplified by PT104, a 35-year-old Filipino man who stated:

*So, like, I—I—for me, it’s not—I don’t feel—I don’t think it’s, like, the chemical high—the fizz, uh, physiologic. I think it’s more just like the habit of, like, smoking—like taking that deep breath and, like, puffing it out. I think that’s more of my, um, addiction, not necessarily [nicotine].*

It was expected participants in this group would mention prototypical nicotine effects such as light-headedness due to a lower tolerance from less use, but some in the low nicotine exposure group reported no longer experiencing these effects—“I don’t buzz hard anymore”. This is consistent with tolerance to the effects of nicotine, which is interesting given the relatively low levels of nicotine exposure among individuals in this group. This mimicked what was reported by participants in the high nicotine exposure group. Conversely, some cases in this group reported experiencing undesirable nicotine effects “vaping doesn’t make me feel good anymore”, which may partially account for low use in this group.

### 3.2. Addiction Narratives 

The level of nicotine dependence was based on participant descriptions of withdrawal experiences as well as attitudes related to quitting, including quit attempts. Addiction narratives among cases in the high nicotine exposure group, regardless of product or products used, were comparable. That is, regardless of whether participants used ENDS, nicotine replacement therapy, combustible cigarettes, or some combination of these products, they all appeared to experience similar nicotine effects and withdrawal symptoms. Likewise, individuals in the high nicotine exposure group described similar strategies to help offset or cope with nicotine withdrawal. While patterns of products used differed by age group, descriptions of subjective effects of nicotine were similar across ages. Below are exemplary cases from each age group. 

PT123 is a 44-year-old White female who reported successfully quitting cigarettes by transitioning to an ENDS. Her stated intention was to quit all nicotine use within 12 months. However, upon reflecting on her stated intention to quit, she stated, “… I might be more addicted to nicotine now. I don’t know whether I am or not, but—than I was before”. Her feeling that she might be more addicted to nicotine after having quit cigarettes was related to her easy access to vaping—she said vaping was more accessible and socially acceptable than smoking cigarettes. She added that she was unaware of just how much nicotine she was using. Her ENDS was refillable, which she reported made tracking her use difficult. She mentioned the nicotine exposure measurement (simply referred to as “biomarker testing” in interviews) as one way to learn about just how much nicotine she was ingesting. She explained:

*I would be really curious about [biomarker results] that. Because I would hate to think that I’m—and I don’t know. I mean, is there really such a thing as more addicted? I don’t know. I mean, I smoke, you know, or vape, rather, you know, quite often during the day. I don’t think about it. I don’t set it down and then leave it in a room and go get it when I want one; whereas, cigarettes, I just left them by the back door. And when I went out to have a cigarette, I had to get up and go do that.*

The question raised by PT123, “is there really such a thing as more addicted?” indicated her reluctance to acknowledge that she could be more addicted now to an ENDS than she was to cigarettes. Participants explicitly and implicitly identified the unforeseen risk of increasing one’s nicotine dependence in the process of replacing smoking with ENDS. Another example was given by PT108, who describes having greater access to nicotine by having initiated ENDS use following combustible cigarette use: “I was going somewhere where you couldn’t smoke at all. So, I thought this would be a perfect—in combination, this [cigarette and ENDS] would be a perfect tool to—in that department”. Reflecting on her use behavior in the interview, PT123 described her use of her ENDS as “nonstop”. She further reflected on how much nicotine was actually entering her system and whether she was more addicted because she “hit” her vape all the time but only took in a little at a time. She wondered whether taking small hits nonstop was better or worse than using a JUUL or smoking an entire American Spirit brand cigarette. 

Another interesting case was PT127, who unsuccessfully attempted to replace cigarettes with a pod vape and ended up using both products. He smoked a pack of cigarettes a day in addition to using two different types of ENDS. His selection of which product to use was context-dependent. Below is an excerpt explaining the consequence of his attempting to use a pod vape to quit smoking:

*I wanted to try the JUUL. It wasn’t doing it for me. I just thought it would do it. I wanted to honestly quit smoking cigarettes and alternately get my nicotine in other ways. I didn’t want to stop consuming nicotine, I just wanted to not smoke cigarettes due to the smell, everything. And if this has like four ingredients in it versus this who we don’t even know what’s in it, at least the ingredients are on the bottle of e-juice. So, I wanted to quit smoking and to me all these devices did was increase my chances of smoking tenfold. …*

In the case of a younger adult (PT064), he stated that while ENDS are good for people who were “looking to get away from cigarettes” as it was for him, he simultaneously labeled this process as one where he “just transferred addictions from cigarettes to JUUL”. Unlike PT123, PT064 had a distinct vision in mind in terms of quitting vaping altogether. He did not question whether he was more or less addicted to nicotine as an ENDS user; he hypothesized that he would be able to give up JUUL more easily than cigarettes because he was successful in quitting cigarettes. To move from using JUUL to fully quitting nicotine without some sort of replacement may or may not be more difficult than quitting cigarettes in the context of JUUL. PT064 explains:

*While I think that JUUL does a great job of doing that, I do think that it can also be just a … a transfer of addiction from one thing to another. Whether it be cigarettes or smokeless tobacco to JUULing, you know. But I think it can be a very useful tool for people that are looking to get away from cigarettes because I know it—it certainly helped me. And once again, I’m one of those people who just transferred addictions from cigarettes to JUUL. But it did, you know, completely cut cigarettes out in the long run. So, were there to come a time when I actually do stop JUULing, I think it would be easier to get off of this than it would have been cigarettes because I’ve done that before. Just nicotine satisfaction is, I guess, achieved using JUUL, and getting away from cigarettes.*

Across the high nicotine exposure group, whether users were strictly using ENDS, combustible cigarettes, or some combination of the two, their reflections on addiction suggested equal dependence on the different types of products.

The majority of cases in the low nicotine exposure group either denied having withdrawal symptoms or simply stated they did not perceive themselves to be addicted to nicotine. This was the case for PT143, a 34-year-old male who used smokeless tobacco as a way to relax or out of boredom, spontaneously offered that he was “not addicted”:

*It’s more just something that I do from time to time, and I really don’t even know why, it’s just something that I’ve done for years and years and years. But, I’ve—I don’t—I don’t have that addictive personality, I am not addicted to it. I told my wife, when we had our second child five and a half years ago, that I would quit, and I did. It was literally just one day, I just—I had half a can and just threw it in the trashcan and never even thought about it again and it didn’t bother me to not do it.*

Signs of addiction were noted in other interviews, though these were not typically as pronounced or severe as those in the high nicotine exposure group. For example, in the low nicotine exposure group, PT104 reported very consistent pod ENDS use throughout the day, initiating use about 50–60 min after waking. He perceived his addiction was to the habit of tobacco use rather than the nicotine itself. Two of the low nicotine exposure cases were labeled as discrepant, meaning their biomarker data did not align with their reported use. In each of these cases, indications of nicotine addiction similar to those expressed by participants in the high nicotine exposure category appeared in the narratives of participants with low levels of nicotine exposure. PT021, a younger adult, exemplified this scenario:

*The withdrawal symptoms, so, yeah, like the—there’s like, I don’t know how prominent this is in the vape culture…but like, uh, in Southern California and here, like I know it’s a thing in California, like we call it like fiending for it…In the past year I’d say I’ve had days like over the summer where like I’ll run out of a bottle of juice and like I’ll run out of like everything in my vape, and I’ll just start to get—I’ll tell myself like, oh this is okay. … I’ll wait until next week to buy a bottle of juice and then end up buying it two hours later, yeah.*

In the low nicotine exposure group, both an absence and a presence of behaviors commonly associated with nicotine dependence were reported in interviews.

## 4. Discussion

This study represents a uniquely detailed examination of younger and older adults recalling their experiences using multiple types of tobacco products in the natural environment, with the addition of quantitative biomarker data to classify and compare qualitative interview results. One important finding from this study was that younger and older adults, though having somewhat different product use profiles (Figure 1), did not differ in their narratives about addiction and experience of nicotine effects. This finding also generalized to product type, with younger and older adults providing similar product use behaviors, addiction narratives, and describing similar nicotine effects whether they used ENDS, combustible cigarettes, or oral tobacco products. Similar descriptions of nicotine effects were also present among individuals in the low and high nicotine exposure groups, suggesting that even at lower nicotine exposure levels, individuals interviewed have some tolerance to nicotine. Another notable finding from this study was that individuals in the high nicotine exposure group who use multiple tobacco products implied or described how the use of several products allowed them continuous access to nicotine. Though, some individuals in the high nicotine exposure group who had successfully switched from combustible cigarettes to another product also discussed having similar or greater access and perceived addiction to nicotine using only a single product (i.e., ENDS) relative to a combustible cigarette.

This study was initially designed to compare and contrast younger and older adults’ reports of nicotine effects and addiction narratives. However, qualitative results from this study indicated that younger and older adults’ narratives were similar, and therefore the qualitative data were subsequently combined for analysis and presented together. Similarly, another qualitative study found ENDS provided comparable nicotine effects and were perceived as equally addictive among younger (defined as 29 years of age or less) and older adults (defined as 30 years of age or older) [20]. One difference between the younger and older adults in this study that is worth noting is the two most prevalent tobacco product use patterns based on past three-day use. The most common patterns among young adults were exclusive ENDS use and exclusive combustible cigarette use, whereas, for older adults, the most common patterns were exclusive combustible cigarette use followed by the dual use of ENDS and combustible cigarettes (Figure 1). This is consistent with research demonstrating that ENDS use is more popular among younger adults (defined as 18–24 years of age) relative to older adults (defined as 25 years of age or older) [21]. Among the high and low nicotine exposure groups, we found that addiction and nicotine effects narratives were similar across age groups. This similarity may suggest that the addiction to nicotine is similar between product types, and individuals using ENDS will not necessarily subsequently transition to combustible cigarette smoking if they are satiated by their current product(s). This may reflect the fact that newer ENDS (e.g., pod-style ENDS) used by many participants in this study were shown to be capable of matching the speed and quantity of nicotine delivery of combusted cigarettes [22]. This transition to smoking is of concern, as a 2021 meta-analysis of 17 studies found nonsmoking young adults using ENDS have four and a half times the odds of subsequent cigarette smoking [23].

New tobacco products may be an alternative to combustible cigarettes, which had long been the primary form of nicotine consumption. While there are no long-term studies to definitively address whether these new products are effective at reducing harm, at least with regards to ENDS, research indicates lower short-term exposure to many toxicants associated with the negative health effects of combustible cigarette smoking among exclusive ENDS users versus exclusive combustible cigarette users [24]. However, there are potential health consequences, such as those associated with nicotine exposure [25], and the physical and psychological effects of addiction and nicotine withdrawal, as well as cardiovascular and pulmonary risks [26,27]. Narratives from interview data in this study indicate that individuals using a variety of different tobacco products have similar addiction narratives. This is somewhat unsurprising as all the products contain nicotine, which is the primary reinforcer in combustible cigarettes [28], and it was likely the goal of many new product manufacturers to develop a product that matches the speed and quantity of nicotine delivery of combustible cigarettes. For example, the ENDS manufacturer JUUL indicates that their product provides comparable satisfaction to combustible cigarettes in a patent establishing the use of nicotine salts in their product [29]. Given the similarity of addiction narratives across products in the current study, future research on the potential tolls of addiction to, and the ability of users to reduce or quit using, new tobacco products are warranted.

It was surprising to note that similar addiction narratives and descriptions of nicotine effects as found in the high nicotine exposure group were also present, though less pronounced, in the low nicotine exposure group. Prior to the analysis of these data, it was speculated that individuals in the low nicotine exposure group might use tobacco products for desirable subjective effects (e.g., stimulating effects of nicotine) or social reasons but not for withdrawal symptom relief, which is consistent with data from light and intermittent cigarette smokers [30]. However, individuals in the low nicotine exposure group reported no longer experiencing desirable subjective effects and using out of habit. There is evidence that regular smokers report greater pleasure from smoking and similar relief from craving to smoke relative to intermittent smokers [31]. As such, it may be that the individuals in the low nicotine exposure group consume less nicotine because they do not find it as pleasurable as individuals in the high nicotine exposure group. It is also worth keeping in mind that individuals in the low nicotine exposure group had used tobacco for at least one year (older adults) or two years (younger adults) since they were recruited into this study. Thus, it may not be how much nicotine they use but how long they have used it that has led to decreased desirable effects and the presence of withdrawal symptoms. Lastly, it should be noted that there is evidence of craving and withdrawal symptoms appearing among adolescent smokers preceding the onset of daily smoking [32], though it is unclear how generalizable the experience of adolescent smokers is to adults. 

A significant concern with non-combustible tobacco products is that they can be used more discreetly than combustible tobacco products. Past research has indicated that approximately two-thirds of adult ENDS users engage in some form of stealth vaping (the act of discreetly using ENDS where they are prohibited [9]). Similarly, adult tobacco users have indicated utilizing smokeless tobacco products in places where smoking is prohibited [33]. Several participants in the current study described having greater continuous access to nicotine after switching from combustible cigarettes to a non-combustible tobacco product. For this reason, one participant further indicated feeling more addicted to an ENDS compared to when they used combustible cigarettes. With regards to smokeless tobacco, research on data from 2013 to 2014 indicated higher levels of nicotine exposure relative to combustible cigarettes [34], which would support the idea that products that can be used more discreetly can lead to greater nicotine exposure than combustible cigarettes. Data on ENDS users, however, did not indicate greater nicotine exposure relative to combustible cigarettes [35]. Similarly, in terms of nicotine dependence, smokeless product users have demonstrated comparable dependence and ENDS users slightly less relative to combustible cigarette users [35]. ENDS, however, have rapidly evolved from products that delivered considerably less nicotine than combustible cigarettes [36] to the most recent salt nicotine-containing products than can meet and exceed the nicotine delivery of combustible cigarettes [37]. As such, future studies may find that nicotine exposure among ENDS users will be increased compared to earlier studies.

The strengths of this study include having a sample of younger and older adult tobacco users using a diverse array of products, having biological verification of nicotine exposure, and collecting detailed information on tobacco product use using a semi-structured interview format. Despite these strengths, there are several limitations the reader should consider when interpreting this research. First, there was an unexpectedly high percentage of recent (past 3-day) cannabis use among participants in this study. Because this study focused on tobacco use, interviews did not focus on cannabis use, and biomarkers of cannabis use were not collected. Second, though adults were categorized as younger or older based on age, the older adult group encompassed individuals ranging from 32 years of age and older, and differences among older adults may exist within individuals in this wide-ranging age group.

## 5. Conclusions

This study combined qualitative narratives and biomarkers of nicotine exposure, finding that both high and low levels of nicotine exposure are reflected in narratives consistent with features of nicotine addiction, regardless of age and specific products used. This suggests that a diverse market of tobacco products provides many options to access nicotine and that dependence on nicotine may persist as a greater variety of tobacco products are offered. Addiction may occur even with low levels of use and non-cigarette products and with both single product and multiple product use.

## Figures and Tables

**Table 1:** Demographic and Baseline Cigarette Use Variables.

	Younger Adults (*N* = 27)	Older Adults (*N* = 20)	*Statistic* (*p*)
**Demographic Variables**			
Age, *mean* (*SD*)	25.6 (3.8)	48.7 (10.3)	***t =* 11.0 (<0.001)**
Gender, *N (%)*			𝜒^2^ = 1.4 (0.491)
Male	22 (81.5)	15 (75.0)	
Female	5 (18.5)	4 (20.0)	
Non-Binary	0 (0.0)	1 (5.0)	
Race, *N* (%)			𝜒^2^ = 2.6 (0.755)
Black	1 (3.7)	2 (10.0)	
Asian	6 (22.2)	3 (15.0)	
White	17 (63.0)	14 (70.0)	
Other	3 (11.1)	1 (5.0)	
Hispanic, *N* (%)	8 (29.6)	3 (15.0)	𝜒^2^ = 1.4 (0.242)
**Tobacco Use Variables**			
Cigarettes/Cigars			**𝜒^2^ = 7.2 (0.007)** *t* = 2.0 (0.055)
Used in past 3-days, *N* (%)	11 (40.7)	16 (80.0)	
Cigarettes per day (Past 3 Days) *, *mean* (*SD*)	6.2 (6.9)	6.8 (4.6)	
ENDS			𝜒^2^ = 2.2 (0.137) *t* = 0.5 (0.647)
Used in past 30-days, *N* (%)	18 (66.7)	9 (45.0)	
Puffs per day (Past 3 Days) *, *mean* (*SD*)	4.6 (5.0)	10.0 (23.0)	
Smokeless Tobacco/Oral Nicotine Products			𝜒^2^ < 0.1 (0.986) *t* = 0.1(0.962)
Used in past 30-days, *N* (%)	4 (14.8)	3 (15.0)	
Uses per day (Past 3 Days)*, *mean* (*SD*)	3.8 (2.9)	3.9 (2.6)	
Cannabis			𝜒^2^ < 0.2 (0.638) 𝜒^2^ < 0.8 (0.358)
Used Combusted in past 3-days, *N* (%)	9 (33.3)	8 (40.0)	
Used Non-combusted in past 3-days, *N* (%)	5 (40.0)	6 (30.0)	

* Average calculated only within individuals who endorsed using this product type in the past 3-days.

**Table 2:** High and Low Nicotine Exposure Demographics and Baseline Cigarette Use Variables.

	Younger Adults (*N* = 27)	Older Adults (*N* = 20)	*Statistic* (*p*)
**Demographic Variables**			
Age, *mean* (*SD*)	33.3 (13.0)	37.0 (13.5)	*t* = 0.6 (0.539)
Gender, *N (%)*			𝜒^2^ = 3.5 (0.060)
Male	7 (70.0)	9 (90.0)	
Female	3 (30.0)	0 (0.0)	
Non-Binary	0 (0.0)	1 (10.0)	
Race, *N* (%)			𝜒^2^ = 5.6 (0.133)
Black	0 (0.0)	0 (0.0)	
Asian	3 (30.0)	0 (0.0)	
White	6 (60.0)	9 (90.0)	
Other	1 (10.0)	1 (10.0)	
Hispanic, *N* (%)	3 (30.0)	0 (0.0)	𝜒^2^ = 3.5 (0.060)
**Tobacco Use Variables**			
Cigarettes/Cigars			𝜒^2^ = 0.2 (0.653) *t* = 1.7 (0.108)
Used in past 3-days, *N* (%)	4 (40.0)	5 (50.0)	
Cigarettes per day (Past 3 Days) *, *mean* (*SD*)	2.0 (3.9)	14.0 (6.6)	
ENDS			𝜒^2^ = 0.2 (0.653) *t* = 1.7 (0.157)
Used in past 30-days, *N* (%)	5 (50.0)	6 (50.0)	
Puffs per day (Past 3 Days) *, *mean* (*SD*)	17.9 (23.7)	190.0 (265.2)	
Smokeless Tobacco/Oral Nicotine Products			𝜒^2^ = 0.0 (1.000) *t* = 0.7 (0.503)
Used in past 30-days, *N* (%)	2 (20.0)	2 (20.0)	
Uses per day (Past 3 Days) *, *mean* (*SD*)	0.3 (2.4)	5.0 (2.8)	
Cannabis			𝜒^2^ = 0.0 (1.000) 𝜒^2^ = 0.4 (0.531)
Used Combusted in past 3-days, *N* (%)	3 (30.0)	3 (30.0)	
Used Non-combusted in past 3-days, *N* (%)	2 (20.0)	1 (10.0)	

* Average calculated only within individuals who endorsed using this product type in the past 3-days.

**Table 3:** Total Nicotine Equivalent Levels.

	Minimum	25th Percentile	Geometric Mean	Median	75th Percentile	Maximum
**Overall Sample**						
Uncorrected, ng/mL	0.25	7.01	15.77	20.65	46.07	97.40
Creatinine Corrected, ng/mg	0.73	10.29	21.18	25.86	44.19	103.02
**Low Nicotine Exposure Group (*N* = 10)**						
Uncorrected, ng/mL	0.25	0.72	1.77	2.20	3.70	15.99
Creatinine Corrected, ng/mg	0.73	4.54	5.15	5.56	9.59	23.18
**High Nicotine Exposure Group (*N* = 10)**						
Uncorrected, ng/mL	51.50	54.38	61.53	57.62	63.93	97.40
Creatinine Corrected, ng/mg	28.10	42.11	52.82	57.66	66.16	103.02

## Data Availability

The data presented in this study are not publicly available due to privacy concerns.
